# Artisanal Brazilian Cheeses—History, Marketing, Technological and Microbiological Aspects

**DOI:** 10.3390/foods10071562

**Published:** 2021-07-06

**Authors:** Ana Lucia Barretto Penna, Mirna Lucia Gigante, Svetoslav Dimitrov Todorov

**Affiliations:** 1 Department of Food Engineering and Technology, São Paulo State University—UNESP, São José do Rio Preto 15054-000, Brazil; ana.lb.penna@unesp.br; 2 Department of Food Technology, State University of Campinas, UNICAMP, Campinas 13083-862, Brazil; mirna@unicamp.br; 3 Department of Food Science and Experimental Nutrition, São Paulo University—USP, São Paulo 05508-000, Brazil; 4 ProBacLab, Department of Advanced Convergence, Handong Global University, Pohang, Gyeongbuk 37554, Korea

**Keywords:** dairy products, geographical indication, cheese quality, cheese microbiota

## Abstract

This review focused on the historical, marketing, technological, and microbiological characteristics of artisanal Brazilian cheese. Brazilian cheese production was introduced and developed from the influence of immigrants considering the combination of climate, races of the animals, quality and specificity of milk, technological cheese-making processes and environmental microbiology, among other factors. It resulted in cheese products with specific physicochemical, microbiological, and sensory quality, which represent the heritage and identities of the different Brazilian regions. The production of artisanal cheese increased in many Brazilian regions, mainly in the southeast, especially due to the traditional production and innovative development of new varieties of cheese. The microbiological quality and safety of raw-milk artisanal cheese continues to be a concern and many studies have been focusing on this matter. Special attention needs to be given to the cheeses produced by raw milk, since numerous reports raised concerns related to their microbiological safety. This fact requires attention and the implementation of strict hygiene practices on the production and commercialization, besides appropriate governmental regulations and control. However, more studies on the relationship between technological processes and microbiological properties, which results in a superior culinary quality and safety of artisanal Brazilian cheeses, are needed.

## 1. Introduction

Latin American fermented food products have been developed from the combination of the folk traditions of native indigenous people and the strong influence of European traditions, generally referring to the practices of Portuguese, Spanish, and Italian immigrants, who conquered South and Central America. Later, immigrants from other European countries and Asia have given their contribution to these fermentation processes based on their own traditional knowledge. Nowadays, Latin American fermented food products can be considered as an amalgam of the broad European knowledge, with influence from Asian food preparation practices, and a touch of African and Indigenous American essence.

Cheese production in Brazil did not really differ from the previously mentioned trends. Most of the cheeses produced in Brazil are influenced by European cheese-making practices, including Portugal, Spain, and non-Iberian countries, such as The Netherlands, Denmark, Greece, France, Italy, and Switzerland [1,2]. The knowledge of these immigrants generated a unique profile of fermented food products in Brazil throughout the last four centuries. Most of the dairy products, including cheese, were prepared in an attempt to reproduce the old-World products in Latin America. However, based on the specificity of the climatic and infrastructure conditions in Latin America, animal feeds, race of dairy animals, raw milk quality, indigenous microbiota, among other factors, it was impossible to reproduce the same European cheese, so new varieties of already known products have been developed [3]. Therefore, the artisanal Brazilian cheeses are unique products and represent the different Brazilian regions’ heritage and identity.

The Brazilian geographical areas developed specific varieties of artisanal cheese which include fresh and ripened cheeses, produced with cow, goat, sheep, buffalo, or a mixture of the milk from different animal species. The production of cheese involves physical, biochemical, and microbial changes in milk, such as enzymatic transformation or acidification, gel coagulation, reduction of water activity, and degradation of milk components. The development of the cheese taste is essentially due to the biochemical transformation of lactose, proteins, and lipids into organic acids (lactic, propionic, formic, acetic, and others), peptides, free amino acids, amines, aldehydes, and methyl ketones, respectively [4]; the intensity of these transformations depends on the ripening time.

The number of studies dedicated to characterizing the historical, technological, and microbiological characteristics of artisanal Brazilian cheese has increased over the last few years, demonstrating their importance. Some papers, theses, and books feature artisanal Minas cheese, such as Canastra [5,6,7], Serro [8,9,10], Campo das Vertentes [11,12,13], Serrano [14,15,16], Coalho [17,18], and Colonial [1,19,20,21] cheeses. Currently, however, Brazil offers a wide variety of artisanal cheeses, generally distributed regionally, which are still little known, even among Brazilians.

In this review, we present the historical overview of the traditional artisanal Brazilian cheeses, the marketing and future of artisanal cheese in Brazil, as well as the characteristics of different types of artisanal cheese, general manufacturing process and regions of production, along with the microbiological aspects and safety of these cheeses.

## 2. Historical Overview of Artisanal Brazilian Cheeses

Cheese is one of the oldest fermented food products of human civilization and although it is not known when it first arose, it probably appeared after the domestication of goats, sheep, and cows. Analytical techniques based on the carbon-13 isotope (δ^13^C) values of the major fatty acids of milk fat applied to pottery vessels found in archaeological sites in Europe evidenced that milk was in use in the seventh millennium Before Christ (BC); this is the earliest direct evidence to date [22]. In Brazil, the practice of using milk from cow, goat, sheep, and buffalo in cheese manufacturing is much more recent and there are few reports in the Brazilian colonial historiography about cheese production [23]. A recent study on the geography of Minas Artisanal cheese revealed that the cheese production in the region of Minas Gerais occurred simultaneously with the occupation of the new territories, establishment of settlements, and gold mining [1,23]. However, the gold rush that led to the Portuguese colonization in Brazil only occurred in the late 17th and early 18th centuries, and the first herds of cattle landed in the captaincy of São Vicente, Brazil, in 1534 [23]. Thus, given the presence of cattle and of Portuguese immigrants who were habitual consumers of cheese, the cheese production in Brazil may have originated in São Vicente. After 1534, many more cows, goats, and horses may have landed on the Brazilian coast, so it is not reasonable to assume that cheese has not been manufactured in Brazil for more than 150 years. According to the Agricultural Research Company of Minas Gerais [24], although there is no scientific research on the geographical and historical origin of cheese manufacturing in Brazil, it is possible that cheese production may have started in 1536, for the intention of using milk and to ensure a greater durability for food.

Despite the lack of historical records, cheese manufacturing has a long history in Brazil and is present in most regions of the country. In the North, in the state of Para, Marajó cheese has been produced for over 200 years in the Marajó Island; in the Southeastern region, Minas Artisanal cheese stands out, certainly the best-known artisanal cheese in Brazil, dating back to the end of the 18th century [25]. The artisanal way of making Minas cheese was considered by the National Institute of Historical and Artistic Heritage (IPHAN) in 2008 as a Brazilian intangible cultural heritage. Its origin, usually attributed to the Serra da Estrela cheese produced throughout the northern central region of Portugal with sheep milk and vegetable coagulant [1], has been questioned. According to Netto [23], the Minas Artisanal cheese has originated from the Pico and São Jorge cheeses, both from the Azorean islands, made with cow’s raw milk and animal rennet, with a ripening period remarkably similar to that of Minas Artisanal cheese.

In the South, the Colonial cheese stands out; it is produced in the Brazilian states of Paraná, Rio Grande do Sul, and Santa Catarina. The immigration of European farming families, especially from Portugal, who colonized these regions, as from the first half of the 19th century, took the form of settlements known as “colonies”, characterized by self-sufficient, diversified farms divided out on an egalitarian basis [26].

The Serrano cheese is also distinguished in this region, with the first record of manufacture in 1831, when small farmers from Rio Grande do Sul requested to the president of the province road improvements for the distribution of regional products, such as cheese and butter, which were exchanged with consumable items [1,27].

The Coalho cheese is the most typical artisanal cheese in the Northeastern region, produced in hinterland farms since Brazil was a colony of Portugal, mainly in the states of Pernambuco, Rio Grande do Norte [17], Alagoas, Ceará, Sergipe, and Paraíba. It is mainly characterized by its resistance to heat and is usually grilled for consumption [26]. Nowadays, for the industrial production of Coalho cheese, pasteurized milk is used, and it is produced in almost all states of the country [1].

The production of goat cheese in the Northeastern region is also reasonably old, mainly because goat farming has been carried out since the Portuguese colonized Brazil, especially in the states of Paraíba and Rio Grande do Norte. Goat cheeses, such as Arupiara, Cariri, Aroeira, and Cabra da Serra, stand out because they are considered a premium type of cheese and have received awards in Brazilian food competitions [1].

Although in Europe, thousands of agricultural products have geographical indication (GI) certificates, in Brazil, this is a quite recent practice. Brazilian GI legislation was initially promulgated as a direct consequence of Brazil’s adjustment to membership of the World Trade Organization (WTO) and the Trade-Related Intellectual Property Rights (TRIPS) agreement in 1996. The former included agriculture in the regulation of world trade and the latter required, as one of its conditions, the adoption of intellectual protection in the case of geographical indications. Two categories of GI were then adopted: indications of provenance (IP) and denominations of origin (DO) [26].

Nowadays, the IP has been granted to three Brazilian artisanal cheeses: Minas Artisanal Serro, Minas Artisanal Canastra, and Witmarsum Colony cheeses. Recently, the Marajó cheese received the first certificate of artisanal product from the Pará state. Additionally, there is an intention to require its GI registration, and this product is also a candidate for immaterial Brazilian heritage by the IPHAN [27]. It is produced by small producers, with different consistencies: more or less creamy or spreadable.

Furthermore, the Association of the Producers of Cheese and Dairy Products from Campos de Cima da Serra, and the Association of the Producers of the Artisanal Serrano Cheese of Santa Catarina Mountain were founded, which was important for the establishment of the Technical Regulation to fix the Identity and Quality of Artisanal Serrano cheese in 2014, as well as for requiring the geographical identification [1,14]. Moreover, the Producers Association of Artisanal Serrano Cheese of the Brazilian states of Santa Catarina and Rio Grande do Sul states requested the National Institute of Industrial Property (INPI) to recognize its DO, which is under analysis.

The WTO agreement on trade-related aspects has recognized the GI as a mechanism that adds value to food products in international trade negotiations. Therefore, the GIs represent an opportunity for the internationalization of small businesses, when applied strategically in the country, and it may not compete with commodities in the international market, but can definitely offer unique, high value-added products [26].

## 3. Marketing and Future of Artisanal Cheeses in Brazil

Brazil is a country of continental dimensions—the production and marketing of small-scale artisanal cheeses, without government support or an organized distribution system, have been restricted almost exclusively to the producing regions for many years. However, in the last decade, the artisanal Brazilian cheese has been receiving a lot of attention, not only due to its traditional production in some regions, but also its innovative production, with high-quality ingredients, creative techniques of production, and introduction of specific animal feed to obtain high-quality milk, especially in some dairy plants in Southeastern and Southern Brazil. In some cases, there are adaptations of the production of international cheeses, yet with the characteristics of the Brazilian milk, pasture, and climate.

The development of this industry is notable and has been driven by the growing consumer interest in natural products and the ongoing transformation of the food standard, which emphasizes the uniqueness of products and the creation of small market niches that enhance the local production capacity. In this context, not only cheeses manufactured according to historical and cultural traditions as those mentioned above, but also an increasing number of new artisanal cheeses have been produced and marketed in the country, e.g., in the states of São Paulo, Mato Grosso, Mato Grosso do Sul, and Goiás.

An important limitation to be faced for the future of artisanal cheeses in Brazil is the legal aspect, especially regarding using raw milk or not in its manufacture. Certain European countries allow the manufacture and sale of soft ripened, semisoft, and hard, aged raw-milk cheeses, while the United States of America demands that cheese made with raw milk could only be marketed after 60 days of ripening. However, in Brazil, the Normative Instruction (IN) 30/2013 of the Ministry of Agriculture, Livestock and Supply regulated and authorized the sale of artisanal cheeses traditionally made with raw milk throughout the country. The traditional Brazilian artisanal raw-milk cheeses could be ripened for less than 60 days, when technical-scientific studies demonstrate that the reduced ripening time does not compromise the quality and harmlessness of the product, besides other requirements [28].

The use of raw milk is a cause of concern because it constitutes an important source of pathogens, such as *Listeria monocytogenes* [29,30], *Staphylococcus aureus* [8,31,32], *Campylobacter* spp. and *Escherichia coli* [6]. Additionally, food-borne infections linked to cheese consumption were reported in many countries, including Brazil [21]. Milk pasteurization, which is also applied to guarantee the sanitary quality of the product, can be obtained through high temperature/short time (HTST) pasteurization (72–75 °C/15–20 s) or equivalent conditions to ensure negative alkaline phosphatase [33]. Although pasteurization reduces the risk of pathogens, it eliminates the autochthonous microbiota, responsible for the characteristic flavor of the most traditional raw-milk cheese products [21,34]. When milk is not pasteurized, it is essential to perform the sanitary control of the herd and have the Food Safety Management System (FSMS) implemented, such as the establishment of Standard Operating Procedure (SOP), the adoption of Good Manufacturing Practices (GMP), and Hazard Analysis and Critical Control Points (HACCP), in order to avoid the risk of pathogen transmission via the consumption of artisanal cheeses. Regarding the microbiological standard for Brazilian cheeses, according to the moisture of the cheese, it is necessary to evaluate the presence of coliforms, thermotolerant coliforms, *Salmonella* spp., and coagulase-positive *Staphylococcus* (for cheese <36% moisture), besides *L. monocytogenes* (for cheese >36% moisture and <55% moisture), and also yeast and molds (for cheese added of lactic acid bacteria (LAB) and >55% moisture); i.e., the required standards vary for each type of cheese [33].

Despite all the scientific knowledge about potential risks, raw-milk cheese products are consumed worldwide and, in Brazil, it is not different. To preserve the traditional and regional methods of production that represent a cultural heritage, the states of Minas Gerais, Santa Catarina, and Rio Grande do Sul have regulated the manufacture of raw-milk cheeses. The Minas Gerais state strategy for GI cheese involves proposals for controlling animal health and redesigning cheese-making facilities and utensils, besides establishing GMP [26]. However, cheese made with raw milk is considered illegal in some states. Today, the raw-milk cheese debate is intensifying, as challenges are being posed to the microbiological safety merits associated with the 60-day rule. There is a general confusion surrounding the scientific basis for permitting raw-milk cheese manufacture, particularly related to aged hard cheeses, and the chemical and composition characteristics which make these products safe. The pasteurization dilemma is, therefore, a complex issue, with profound implications for the future of global trade, public health, and artisanal cheese-making [35].

## 4. Characteristics of Brazilian Artisanal Cheeses, General Manufacturing Process, and Regions of Production

**Figure 1:**
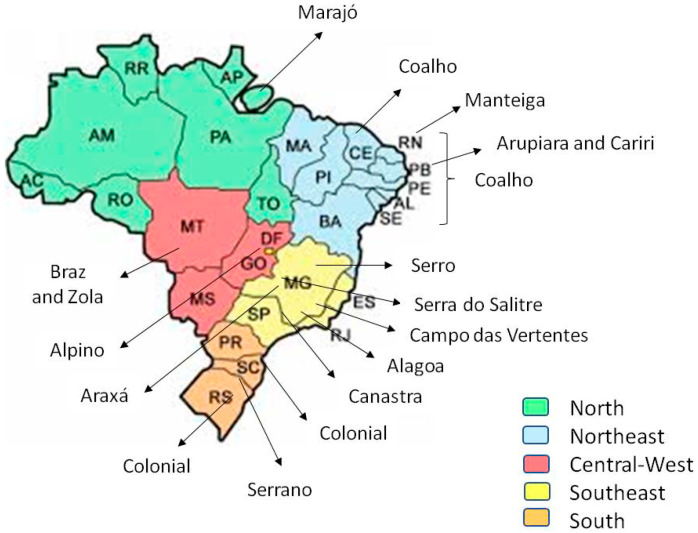
Different cheeses and main production regions in Brazil.

The typical artisanal Brazilian cheese presents a unique quality, related to its traditional processing and to the characteristics of its raw materials and ingredients. Cheese products can be divided by regions, as illustrated in Figure 1. In the North, Queijo do Marajó is the best known. In the Northeast, there are Arupiara, Arupiara Real, Cariri Temperado, Coalho, and Queijo-Manteiga. In the Central-West region, Alpino, Boursin de Cabra, Braz Mato-Grossense, and Zola Mato-Grossense are the most important cheeses. Tulha, Cuesta Azul, Azul do Bosque, Cabacinha, Coração em Brasa, Embriago d’Alagoa, D’Alagoa Faixa Dourada, Minas Artisanal, Pirâmide do Bosque, Giramundo, Simental, and Tropeiro are some typical products from the South-East, and can be distinguished among other cheeses for their industrial production. In the South, the production of Azul de Ovelha, Colonial, Curado and Extra Curado de Ovelha, and Serrano are the most traditional ones. Many other artisanal cheeses are produced; however, they have not become as popular because their production is small and their availability is limited to a very local niche market.

Additionally, to achieve more efficiency, quality output, and uniformity of performance, as well as to reduce failures and achieve a sustainable market, an important issue in artisanal cheese production is the necessity of establishing SOP and using GMP, as previously mentioned. Furthermore, in general, there are few studies on Brazilian artisanal cheeses and almost all of them focus on their microbiological characteristics; on the other hand, their physicochemical composition, their manufacturing technology and/or sensory properties have been sparsely studied. Below we present the characteristics of the best known artisanal Brazilian cheeses.

### 4.1. Marajó Cheese

This cheese is typically produced in the Marajó Island with skimmed raw buffalo milk and is consumed without ripening; however, it was originally produced using cow’s milk [25]. Marajó cheese was produced artisanally until 2001, when a semi-industrial dairy plant was installed in the region. Some dairy plants introduced the GMP, milk pasteurization, and the use of commercial starter cultures; however, these changes resulted in altered sensory characteristics compared to the original product. This cheese is classified as medium humid (36.0% to 45.9% moisture) and fat (45.0% to 59.9% fat in dry matter), according to the Technical Regulation for Identity and Quality of Cheese [33]. The sensory characteristics also include: slightly acid and salty taste, and slightly rubbery and very soft texture [36].

Marajó is a cylindrical or brick-shaped cheese and usually has about 250 to 500 g. It is produced through spontaneous coagulation (acidification) or by using fermented whey as a natural starter, with cutting, curd treatment and heating, and whey removal [36]. The curd is deacidified using water or buffalo milk, followed by draining, salting, addition of dairy cream or butter, mixing, and cooking (melting, 80 °C/15 s). The cheese is soft, has a compact and closed texture, with small and few pores; it has a pleasant aroma, greenish yellow color and a slightly acid and salty taste [1,37,38].

Marajó cheese presents lower ash and protein content, and higher fat content and monounsaturated fatty acid level, if compared to other Brazilian artisanal cheeses; consequently, it has lower atherogenic and thrombogenic indices and higher desired fatty acids, such as palmitoleic and oleic [39,40]. Additionally, Marajó cheese’s microbiota presents a high prevalence of *Enterococcus* spp. which may be related to the curd cooking step applied in their processing, in which the temperature ranges from 40 to 60 °C. *Enterococcus* spp. can resist for 30 min at 60 °C and is considered a natural component of the indigenous microbiota in Brazilian artisanal cheeses [41].

### 4.2. Arupiara and Cariri Cheeses

These cheeses are typically produced in Taperoá, Paraíba state, with goat’s milk from the Moxotó race, which is traditional and autochthone from this region. The Arupiara cheese is creamy, with strong and intense goat aroma. It is a cylindrical-shaped cheese, has 9 cm diameter and 4.0–4.5 cm height, and weighs about 250 g. It is mild, presents an intense lactic aroma, besides a sweet and slightly spicy taste. It is produced through rennet coagulation, curd heating, whey removal, and 4–5 months ripening. The inner is soft, and the rind is firm [27,42]. The Cariri cheese, in turn, receives some seasonings, such as cumaru (*Dipteryx odorata*), alfazema de periquito (*Alternanthera ficoidea*) or aroeira peper (*Schinus terebinthifolia*). Its shape is cylindrical, with 8–10 cm diameter, 5–4 cm height, and weighs about 250 g. This cheese presents a typical taste and aroma due to the addition of herbs [1].

### 4.3. Manteiga (Butter) Cheese

The Manteiga cheese, also known as Requeijão do Norte, is a typical cheese produced in the region of Seridó, in the states of Rio Grande do Norte and Paraíba. It is a brick-shaped cheese and less frequently cylindrical; the weight varies from 2 to 10 kg. It is produced with skimmed, semi-skimmed, or whole raw cow’s milk by spontaneous coagulation or direct acidification using organic acids; the curd is cut, and heat-treated (55 °C), whey is drained and washed with water and/or milk, salted and heat-melted (85 °C/15 min) with the addition of artisanal melted butter, and then it is molded. The ripening period varies from 1 to 7 days [1,27,38]. This cheese is consumed fresh as an appetizer, paired with sweet desserts, or used as an ingredient in recipes.

### 4.4. Coalho Cheese

Coalho cheese is a typical Brazilian cheese produced in 44 cities from the Northeastern region. The industrial production (type A) takes place mainly in the states of Minas Gerais, Paraná, Pará, and São Paulo. Pernambuco state is requiring the GI registration for the product. This cheese has a particular socio-economic and nutritional importance for the region due to its marketing and consumption [17,27].

The cheese shows heat resistance and is consumed either in its natural form, roasted or grilled. Its shape is square, with 20–22 cm length, 12–13 cm width, and 3.1–3.9 cm height. The Coalho cheese is produced with raw or pasteurized cow’s milk by rennet coagulation, cutting and heating the curd, draining, molding, pressing and salting in brine and cutting; it is usually placed on skewers, in order to be grilled, then it is packed and stored cold. It presents a cream to milky color, has a flat surface, well defined borders, firm consistency with light resistance to pressure and curd without eyes or cracks; its texture is firm, soft and rubbery; it is slightly acid and salty, with dairy fermented and animal flavors [1,17,43].

The gross composition (g/100 g ± SD) of Coalho cheese includes 42.5 ± 4.0 moisture, 23.8 ± 2.9 protein, 25.6 ± 3.5 fat, 4.02 ± 0.69 ash, 4.12 ± 0.38 carbohydrates, and 44.4 fat in dry matter. Regarding the fatty acids (FA, in mg/100 g ± SD of total fat), the cheese shows 145.3 ± 0.3 short-chain saturated FA, 172.0 ± 0.5 medium-chain saturated FA, 356.7 ± 0.4 long-chain saturated FA, 212.0 ± 0.3 monounsaturated FA, 117.3 ± 0.3 polyunsaturated FA, 2.0 ± 1.0 atherogenic index, 2.8 ± 0.8 thrombogenic index, 463 ± 9 desirable hypocholesterolemic FA and 378 ± 6 hypercholesterolemic saturated FA [39]. This cheese is consumed fresh as an appetizer, paired with sweet desserts or used as an ingredient in recipes [1].

### 4.5. Artisanal Minas Cheese

Artisanal Minas cheeses are undoubtedly the most well-known and studied artisanal cheeses in Brazil, with seven producing areas: Araxá, Campo das Vertentes, Canastra, Cerrado, Serra do Salitre, Serro, and Triângulo Mineiro. They are produced by farmers with the whole raw bovine milk on a small scale, rennet, salt and by manually pressing it, which results in cheeses with different sensory, physicochemical, and microbiological characteristics. Santos et al. [44] provided a compilation of data available in the literature on the characterization of the cheeses from the different areas of the Minas Gerais region, gathering data on pH, fat content, fat in the dry extract, acidity (g/100 g lactic acid), moisture, sodium chloride content, protein, ripening extension index (REI), ripening depth index (RDI) of Serro, Canastra, and Araxá cheeses. Using artificial neural network (ANN) and linear discriminant analysis (LDA), the authors contrasted the cheeses from different origins according to RDI, REI, and pH. It is usually consumed as an appetizer or used in the typical Brazilian recipe of *pão de queijo* (cassava cheese puffs).

#### 4.5.1. Serro Cheese

The Serro cheese is considered a cultural heritage in Brazil, produced over the last 200 years in the Serro region. Throughout this period, its manufacture underwent some changes, such as type of natural lactic starter, ripening conditions, and salt content. It is generally accepted that its production lacks standardization, which results in the production of cheeses with different sensory characteristics [9,30].

It is traditionally produced with raw bovine milk, the addition of a natural fermentation starter (autochthonous cultures, known as *pingo* and *rala*), and industrial liquid rennet for coagulation. Traditionally, *pingo* is a natural starter produced by salted and fermented whey drained from the cheese produced in the previous batch; in turn, *rala* is the artisanal cheese grated up to 8 days of ripening [9]. It can be consumed fresh or ripened, the curd is homogeneous and consistent, without eyes or gaps, its rind is thin, and the inside is smooth. This cheese is very popular in Brazil and is a diversification of a semi-hard cheese because it has a typical, markedly acidic taste [45].

This type of cheese is normally ripened for 3 to 15 days at room temperature and then stored at 10 °C. It has a cylindrical shape, with 13–15 cm diameter, 6–8 cm height, 0.7–1.0 kg, fine rind, without cracks; its curd is yellowish-white, it has a firm texture, semi-hard consistency, and is more humid than the other artisanal Minas cheeses; the taste is mild and slightly acidic. In general, it presents 46–52% total solids and a minimum of 50% fat in dry matter [10,12,45,46].

The different starter cultures traditionally used in cheese production (*pingo* or *rala*) do not affect the physicochemical properties (pH value, acidity, moisture, and salt contents) of the products. Salt-in-moisture does not differ from one starter culture to the other, nor ripening conditions (3 to 31 days under different temperatures and packaging). In contrast, sensory acceptability of Serro artisanal cheese is greatly affected by the ripening conditions, because organoleptic properties changed as maturation time was extended and influenced by process conditions. Vacuum-packed cheese ripened at 8 °C was almost three times more likely to be accepted than unpacked cheese ripened at room temperature [9].

#### 4.5.2. Canastra Cheese

The typical Canastra cheese is a popular artisanal Brazilian cheese, produced in the area of the Canastra Mountain, in Minas Gerais. The combination of several factors and the cheese-making knowledge originally from immigrants has resulted in the creation of this unique Brazilian cheese. Normally, most of the producers claim that no starter cultures are used in the production of this kind of cheese; however, it is produced using fermented whey as a natural starter (known as *pingo*), added further on during the cheese-making process. This procedure can be considered as enrichment with some LAB already existing in the previous preparation. Additionally, it must be ripened for at least 22 days at room temperature since it is produced with raw milk (non-pasteurized milk), and it could be commercialized only in the Minas Gerais state of Brazil. In the past, these cheeses were commercialized by ambulant vendors or at small, family-run shops [1].

In 2002, the Minas Artisanal Cheese Program (*Programa Queijo Minas Artesanal*—PQMA) was created for the standardization of manufacturing processes, packaging, and marketing of artisanal Minas cheeses. Under the registration in the PQMA, the cheeses produced in one of the seven cities of the Canastra Mountain area can be marketed as Canastra artisanal Minas cheese [47].

More recently, the Canastra cheese was considered as a culinary treasure and the interest in this type of cheese has been increasing; it is coveted by consumers and chefs from around the country, since it received several prestigious international awards in 2015, 2017, and 2019, such as the Super Gold, Gold, Silver and Bronze Awards in the *Mondial du Fromage et des Produits Laitiers*, in France [47]. The cheese, Estância Capim Canastra, produced in São Roque de Minas, was the first Brazilian cheese to receive a medal in this championship. Nowadays, the Canastra cheese can be found at supermarkets in other states of Brazil, including São Paulo and Rio de Janeiro states.

It presents a cylindrical shape, with 15–17 cm diameter, 4–6 cm height, and weighs around 1–1.2 kg. Cheese is produced with raw milk using fermented whey as a natural starter (*pingo*) and rennet for coagulation. Its curd is cut, stirred, molded, salted, and ripened for 8–30 days. It presents a yellowish, fine rind, without cracks; the curd is yellowish-white, has a firm texture, semi-hard to soft and buttery consistency; the taste is slightly acid, not pungent, and pleasant [1,48].

The evaluation of Canastra cheese’s gross composition (g/100 g ± SD) shows 41.7 ± 6.7 moisture, 23.7 ± 2.4 protein, 27.0 ± 4.7 fat, 4.00 ± 0.76 ash, 3.57 ± 0.42 carbohydrates, and 46.3 fat in dry matter. The fatty acids profile (FA, in mg/100 g ± SD of total fat) shows 162 ± 0 short-chain saturated FA, 171 ± 0 medium-chain saturated FA, 410 ± 1 long-chain saturated FA, 174 ± 0 monounsaturated FA, 132 ± 1 polyunsaturated FA, 2.4 ± 1.0 atherogenic index, 3.5 ± 0.9 thrombogenic index, 458 ± 10 desirable hypocholesterolemic FA, and 391 ± 7 hypercholesterolemic saturated FA [39].

The sensory profile of Canastra artisanal Minas cheese has been evaluated by Nogueira et al. [49], who considered the influence of different types of packaging (vacuum, nylon mesh, butter paper, polyvinyl chloride (PVC) film, without packaging, and two control samples: PREMAT—the sample with 14 days of maturation, which was evaluated soon after the acquisition on the property and POSTMAT—cheese with 22 days of maturation without packaging, and associated to the main bacterial groups present in the cheeses under each type of packaging. The cheese samples of PREMAT and butter paper packaging were the preferred ones; however, all samples had good acceptance indices, under the attributes of sour flavor, mild flavor, buttery flavor, farm flavor, salty, cured, compacted, and soft. The microbiota of the preferred cheese was composed of *Lactococcus* spp., *Corynebacterium* spp., *Lactobacillus plantarum*, *Streptococcus* spp. (the authors of the present review paper believe that this is *Str. thermophilus*), *Lactobacillus brevis*, and *Lactobacillus casei*. Rodrigues et al. [50] investigated the behavior and consumer perceptions of artisanal Minas cheese after 22 days of maturation using the diary method to collect sensory data (23 sensory characteristics, 2 affective terms, and 8 feelings of expressions) during 10 days. The sensory profile, the behavior, and perceptions of consumers can help the production plan, marketing, and commercialization strategies.

#### 4.5.3. Araxá Cheese

The Araxá cheese has been produced since the 18th century in the area of the Araxá Plateau, which includes 9 cities. It is produced with fermented whey used as a natural starter and rennet for coagulation. The curd is cut, stirred, molded, pressed, salted, and ripened for 22 days. It is less ripened and salty, and milder compared to the Canastra cheese. It is cylindrical, with 14–17 cm diameter, 4–7 cm height, and weighs 1–1.4 kg. The cheese presents yellowish fine rind, without cracks, the curd is white-creamy, has a firm texture with few holes, semi-hard to soft and buttery consistency; the taste is slightly acidic, not spicy, and pleasant [1,27].

In its gross composition (g/100 g ± SD), the Araxá cheese contains 40.1 ± 2.1 moisture, 23.7 ± 1.3 protein, 27.3 ± 1.3 fat, 3.75 ± 0.46 ash, 5.04 ± 0.51 carbohydrates, and 45.7 fat in dry matter, according to Margalho et al. [39].

#### 4.5.4. Campo das Vertentes Cheese

This cheese started to be produced in the 18th century by Portuguese immigrants [1] in the Campo das Vertentes area, which includes 14 cities. It has a cylindrical shape, with 10–17 cm diameter, 3.5–5.0 cm height, and weighs 0.75–0.9 kg. It is produced with fermented whey as a natural starter and rennet for coagulation. The cheese is turned during brining, ripening, and rinsing. It is ripened for 21–30 days; however, the taste is enhanced after 90 days of ripening. It has 5.0–5.3 pH, 2.4–2.83% salt, 50.8–55.3% fat in dry matter (fatty cheese), and 32.6–39.0% moisture (semi-hard to hard cheese) [1,13].

#### 4.5.5. Serra do Salitre Cheese

The cheese produced in the area of the Salitre Mountain is characterized by a creamy and mild taste. This region formally belonged to the Cerrado region; however, because of its high-quality characteristics, the cheese produced there gained prominence. Its intense flavor is obtained during ripening, and the cheese can be found in the market at different ripening stages: fresh, semi-ripened (14–20 days), and ripened (4 months) [1].

The Serra do Salitre cheese is produced using fermented whey as a natural starter and rennet for coagulation; the curd is cut, stirred, molded, and salted. It has a cylindrical shape, with 15–17 cm diameter, 4–6 cm height and weighs 1–1.2 kg; it has a yellowish fine rind, without cracks, the curd is yellowish-white, has a firm texture, semi-hard to soft and buttery consistency; the taste is slightly acidic, not pungent, and pleasant [1,51].

### 4.6. Serrano Cheese

Serrano cheese is a typical product from the Southern region, which includes 18 cities in the area of Campos de Lages, in the state of Santa Catarina, and 11 cities in the area of Campos de Cima da Serra, in the state of Rio Grande do Sul. The region has specific characteristics, such as the presence of native grasslands, high altitude, and well-defined seasons throughout the year, with low temperatures in winter when there is frequently frost and occasionally snow [14].

In general, despite some small differences between the two areas, the artisanal Serrano cheese is produced in small scale by using raw full-fat cow’s milk, recently milked, and rennet for coagulation; the curd is coarsely broken, heated with hot water (75–85 °C), mechanically or manually molded and pressed, and low salt content is added, which results in a mild taste that enhances during the ripening period at <18 °C varying from 20 days to 1 year. The consistency is firm, the curd is uniform, without colorants or preservatives, with or without mechanical eyes, and typical color and taste. Serrano cheese is produced in a brick shape, and weighs 2–3 kg [14,52]. It is considered to be inspired by Parmesan cheese and can be consumed as an appetizer or as an ingredient in sandwiches [1].

Even though some Serrano cheeses can be ripened, most consumers prefer to consume this cheese after about 15–30 days after production, when its main characteristics are: intense taste, semi-hard curd and yellowish color [14]. Its gross composition (g/100 g ± SD) is 39.2 ± 2.1 moisture, 25.0 ± 1.3 protein, 28.1 ± 2.4 fat, 4.02 ± 0.38 ash, 3.64 ± 0.42 carbohydrates, and 46.3 fat in dry matter [39].

### 4.7. Colonial Cheese

Milking is usually carried out two times during the day in cheese production; the afternoon milk is refrigerated, stored, and mixed with the next day’s morning milk. The Colonial cheese is produced with raw or pasteurized milk; the curd is not heated, then it is pressed and ripened for 7 days to 1 year, but the usual ripening period is 30 to 75 days. It has a cylindrical shape, 0.7 to 5.0 kg weight, yellowish-white color, hard or semi-hard texture crust; the inner is soft, with or without eyes, and the consistency is medium; it melts easily when heated. The taste is mild, slightly salty, medium spicy, with fermented dairy and dry fruit flavors. For ripening, the cheese can be covered with wine, pork fat, colorant, or pepper [1,27]. It is considered to be a versatile cheese since it can be consumed fresh as an appetizer, fried and paired with bread, or used as an ingredient in Italian dishes, such as pasta or pizza [1].

The Colonial cheese has, in its gross composition, (g/100 g ± SD) 38.9 ± 4.7 moisture, 24.6 ± 1.6 protein, 28.4 ± 2.4 fat, 3.94 ± 0.31 ash, 4.14 ± 0.50 carbohydrates, and 46.5 fat in dry matter according to Margalho et al. [39].

One interesting finding was that some LAB isolated from some Colonial cheese obtained in the western region of Paraná presented bacteriocinogenic properties able to inhibit spoilage and pathogenic microorganisms, such as *L. monocytogenes*, *Bacillus cereus*, and *Pseudomonas fluorescens* [20]; consequently, they can help increase the cheese safety.

### 4.8. Other Cheeses

More recently, the demand for artisanal cheese with typical characteristics has been increasing and speeding up the production of many products inspired by European cheeses; however, they are receiving new names, based on the specific raw material, climate conditions, race and feed of animals, which result in many cheese variations. Some of their characteristics are summarized in Table 1.

Some cheeses have received awards in competitions. Although they do not have the same historical recognition and traditional technology as the European ones do, they could be the basis for the development of new products for the current demanding gourmet market. Most of these artisanal cheeses are consumed in restaurants, gastropubs, or gourmet markets in many state capitals of the country or in big urban centers.

## 5. Microbiological Aspects

Although the microbiological quality of artisanal cheeses does not depend exclusively on the pasteurization of milk, this is undoubtedly the main controversy surrounding the safety of these products. Pasteurization guarantees the safety of cheeses because it eliminates (or significantly reduces) all potential pathogenic bacteria which could be present in the raw milk. On the other hand, it significantly reduces the presence of natural microorganisms found in milk (beneficial microbiota), which are associated with the typical characteristics and flavor of artisanal cheeses made with raw milk. Raw milk can be considered as a source of beneficial microbes as well. The sanitary control of the herd, the standardization of the manufacturing process, and the Food Safety Management System (FSMS) implementation are possibly the support of the quality and safety of cheeses made with raw milk. The reduction of pH, removal of water, and addition of salt during cheese manufacturing, whether made from raw or pasteurized milk, are conditions that favor the safety of cheeses.

In spite of this, cheeses produced with raw milk may constitute a suitable medium for the growth of pathogens often associated with food-borne diseases. Additionally, the lack of standardization in manufacturing, especially regarding the time of coagulation, natural starter used, curd treatment, pressing, and salting, affect the composition of the final product and, consequently, its safety. Thus, in the market, it is possible to find artisanal cheeses from the same variety with different sensory, physicochemical, and microbiological characteristics [55,56].

In relation to the microbiological aspects of Brazilian artisanal cheese, most of the studies are concentrated on artisanal Minas cheese, probably because of its economic significance. In contrast, studies about the microbiological quality of milk and other artisanal cheese produced in Brazil are scarce. Moreover, most of the studies related to artisanal Minas cheese focus on some specific producers and it is frequently very difficult to draw conclusions about the microbiological composition of beneficial and spoilage microorganisms, including some pathogens.

Different LAB strains isolated from Brazilian cheeses were evaluated and proposed/classified as beneficial organisms as well as confirmed to be able to provide health benefits to consumers by producing antimicrobial metabolites and contributing to the safety of the dairy products [20,57,58,59], expressing some probiotic properties [6,40,60,61], or contributing to the reduction of the allergenicity in some milk proteins [62].

In the last few years, interest in the artisanal cheese microbiota has been increasing worldwide. The milk quality, the various stages of the cheese-making process, the washing water, and cheese processing facilities harbor distinct and complex bacterial communities. The presence and growth of a variety of microorganisms are responsible for the production of many bio-compounds with sensory and antimicrobial characteristics in these cheeses, or can cause spoilage and raise safety concerns.

Perin et al. [63] reported that *Lactobacillus* spp. were the most prevalent ones in the Minas artisanal cheese varieties, such as Serro, Canastra, Serra do Salitre, Araxá, and Campo das Vertentes. In these cheeses, *Lb. garviae, Lb. acidipiscis, Lb. paracasei, Lb. brevis, Lb. plantarum, Lb. curvatus*, *Lb. rhamnosus*, *Lb. fermentum*, *Lb. delbrueckii*, *Lb. buchneri*, *Lb. parabuchneri*, *Lc. lactis* subsp. *lactis*, *Str. thermophilus*, *Pediococcus acidilactici*; *Leuconostoc mesenteroides*, *Weisella paramesenteroides*, *Enterococcus faecalis*, *Kocuria kristinae*, *E. coli*, and *Staphylococcus* spp. were identified.

In another study, Resende et al. [64] recorded the presence of *Lb. rhamnosus*, *Lb. casei*, and *Lb. plantarum* as the main species isolated from milk and Canastra cheese; while Borelli et al. [55] found *Lb. brevis*, *E. faecalis*, *P. acidilactici*, and *W. paramesenteroides*. These differences could be associated with the milk quality and conditions of cheese production. In the artisanal cheese produced in Serra do Salitre, the most frequently identified LAB were *Lc. lactis*, *Enterococcus* spp., and *S. agalactiae* [51]. The presence of *E. faecalis* is associated with poor hygiene conditions during cheese production and *S. agalactiae* is an important pathogen associated with mammary glandule inflammation. These findings raise alerts about the safety of the cheese produced with raw milk and especially those ripened over a short time.

Recently, Kamimura et al. [38] carried out a large-scale mapping of the microbial diversity of 11 different types of artisanal Brazilian cheeses (Marajó, Manteiga, Coalho, Caipira, Araxá, Campos das Vertentes, Canastra, Serro, Colonial, and Serrano). The results revealed clear geography-based and technology-based differences in the cheese microbiota. There was a dominance of LAB and the core microbiota of artisanal Brazilian cheeses included at least six different genera: *Streptococcus* spp. was dominant in Marajó, Cerrado and Serro cheeses. In Manteiga and Coalho cheeses, *Leuconostoc* spp. prevailed, and in Araxá, Campo das Vertentes, Canastra, *Lactococcus* spp. was the most abundant. In addition, the presence of *Erwinia* spp., *Psychrobacter* spp., and *Staphylococcus* spp., which is potentially harmful, was mainly observed in Coalho, Manteiga, and Caipira cheeses produced in the Northern, Northeastern, and Central regions. In contrast, a low incidence of *Staphylococcus* spp. was found in the core microbiota of Araxá, Canastra, and Serrano cheeses produced in the Southeastern region.

The microbiota of the Serro Minas artisanal cheese is mainly composed by LAB, and *Lactococcus* is the most frequently identified genus, followed by *Streptococcus* and *Lactobacillus* [65]. However, the presence of *Streptococcus* spp. needs to be discussed with high attention, since only a few representatives of these genera are considered as safe.

The molecular identification of isolated LAB from the Campo das Vertentes cheese, water, raw milk, and endogenous starter culture showed that *E. faecalis* was the most frequently (42.86%) isolated LAB in cheese samples, followed by *Lc. lactis* (28.57%) and *Lb. plantarum* (14.29%). Additionally, *Lb. brevis* (5.88%), *E. pseudoavium* (5.88%), *E. durans* (5.88%), and *Aerococcus viridans* (5.88%) were isolated from endogenous starter cultures and are described for the first time in the literature [13].

The presence of different LAB can be related with the specificity of this bacterial species metabolism. The most important ability is to metabolize lactose; however, the capacity to grow at low pH, with high salt concentrations, and the temperatures required in cheese preparation and maturation, along with the ability to compete with some fungal species and/or some bacterial starter cultures, are factors that can influence the presence of LAB in the final products.

Some specific metabolic characteristics of LAB can play a crucial role in the consequent presence of LAB in the final products. *Enterococcus* spp. has been isolated from Canastra and other types of cheese very frequently, given the high salt tolerance of these species and their ability to grow even at temperatures around 15 °C [66].

Microbiological analyses of artisanal Minas cheeses were carried out by aiming to show if there was a health hazard of consuming artisanal cheeses, as well as to find out if these kinds of food products could be considered as a source of beneficial microbes. When artisanal cheeses from Cerrado, Araxá, Serro, and Canastra were analyzed by Denaturing Gradient Gel Electrophoresis—Polymerase chain reaction (DGGE-PCR), the species *Str. thermophilus*, *Str. salivarius*, *Lc. lactis*, and *Lb. plantarum* were identified. According to the DGGE-PCR, *Streptococcus* spp. can be considered as predominant in the studied cheeses [11]. However, such a result could lead to different discussions. On the one hand, if we acknowledge the fact that only *Str. thermophilus* has a well-accepted Generally Recognized as Safe (GRAS) status among all the different members of the *Streptococcus* family, then future studies could be performed in order to show if this species really is the predominant *Streptococcus* spp. species found in the Minas cheese. It would be an excellent recommendation to consider this kind of cheese as a beneficial food product, since several studies have shown the role of different *Str. thermophilus* strains as LAB with beneficial properties, including probiotics, bacteriocin production, proteolytic activity, etc. [67]. On the other hand, in the case that other *Streptococcus* species were present and predominant, this can raise an alert as of a potential hazard, since some different species from *Str. thermophilus* of the *Streptococcus* genera are considered to be potentially pathogenic [68].

As an example, Andrade et al. [48] reported *Lb. plantarum* D27 and *Lb. rhamnosus* B25 strains isolated from Canastra cheese with potential probiotic properties. The presence of these two species was not a surprise, since it was reported to be part of the Canastra cheese microbiota [63]. The authors have explored different aspects of the behavior of these two strains in order to evaluate their probiotic potential, as well as to show their survival and presence in the final product. Costa et al. [60] studied eleven *Lactobacillus* spp. and one *W. paramesenteroides* strains isolated from Canastra cheeses in relation to their potential application as probiotics. Isolated strains showed different levels of covering criteria to be accepted as potentially probiotic; moreover, most of them can be considered as potential candidates for future investigations using in vivo tests. However, concerns of these strains (reported by Andrade et al. [1] and Costa et al. [60]) can be really applied as adjunct cultures in the Canastra cheese production such as, if they will have the appropriate technological properties, then what influence will they have on the safety of the food product? Additionally, will they promote health modulating properties for the consumers?

The presence of LAB and yeasts in artisanal Minas cheeses can be related to several factors, among them the fact that the manufacturing practice involves the addition of fermented whey drained from the previous cheese production to be used as natural whey cultures to produce cheese (*pingo*). This practice is similar to that used to produce several other cheeses made of raw milk, such as Parmesan cheese. For Parmesan, this natural whey starter culture contains a high amount of LAB strains even from different species and can vary widely among farms as well as within each cheese facility throughout the year [69,70].

*Kluyveromyces lactis*, *Torulaspora delbrueckii*, and *Candida intermedia* were detected in the Canastra cheese. *K. lactis* and *T. delbrueckii* are known to be involved in the fermentation of lactose and can be responsible for the production of ethanol and numerous aromatic volatile compounds, including 3-methyl-1-butanol, octanoic acid, and ethyl decanoate, important for cheese aroma and flavor [1]. Additionally, yeasts are a significant part of the cheese microbiota, due to their lipolytic and proteolytic activities, fermentation of lactose, production of flavors, tolerance to high salt concentrations, low pH, low water activity, and low temperatures [55,71].

*Debaryomyces hansenii*, *K. lactis*, *Kodamaea ohmeri*, *T. delbrueckii*, *K. marxianus*, *Yarrowia lipolytica*, as well as several *Candida* species, were reported as the most frequent yeasts in whey, curd, and cheese from Canastra facilities [1,55]. Samples of natural starter, cheese curd before salting, and cheese had general yeast counts between 1.7 and 7.9 log CFU/g and varied a lot among different producers. Most of these yeasts are able to produce proteases, lipases, and β-galactosidase and, this way, they have an essential role in the ripening processes of the Canastra cheese. The production of enzymes by yeasts during cheese ripening is directly related to the development of the characteristic flavor and aroma [55].

Cardoso et al. [45] reported that the predominant species in the Serro cheese were *D. hansenii*, *K. ohmeri*, and *K. marxianus*. In the same study, it was shown the beneficial technological properties of some isolated yeasts: *D. hansenii* 28.12 showed low lipolytic and high proteolytic activity; *K. marxianus* 83F and 60P demonstrated lipolytic and β-galactosidase activity, respectively, and *K. ohmeri* 88A had low lipolytic and β-galactosidase activity. The Serro cheese has a complex microbiota associated with raw milk and cattle management, and includes LAB from the genera of *Lactobacillus*, *Lactococcus*, and *Streptococcus*, which contributes to the unique cheese properties and sensory characteristics. This microbiota also includes a large diversity of yeasts which are important for the development of cheese flavor. *D. hansenii* is one of the most common species isolated in artisanal cheeses [29,45].

Regarding contaminations, the presence of total coliforms in raw-milk cheese has been reported in several studies [19,21]. Their presence can be considered an alert for potential fecal contaminations, most probably related to poor manufacturing practices. These contaminations can be related to non-adequate practices in milking, storage, and/or transportation of raw milk, but can also be related to poor conditions during the cheese-making process and inadequate manipulation of the cheese.

Carvalho et al. [21] evaluated the quality of the Colonial cheese produced in 12 rural properties of Serra, a city in the state of Santa Catarina. Many of the samples did not comply with the microbiological standards and did not conform to the current Brazilian regulatory parameters. Additionally, cheese makers did not meet the requirements for producing artisanal cheese made of raw milk. Risk analysis studies should be performed to better understand the real sanitary risks; corrective actions leading to tangible health improvements for the herd and the quality of water, milk, and cheese were recommended.

The presence of coliforms, pathogens, and sanitary indicators in artisanal Minas cheeses (Serro, Canastra, Serra do Salitre, Araxá, and Campo das Vertentes) has been evaluated in many studies. Perin et al. [63] characterized the microbial diversity composition and geographical distribution of artisanal Minas cheese focusing on the characterization of its autochthonous LAB microbiota. The microbial counts varied among cheese samples. Based on the culture, depending on the approach, 93.3% cheese samples were positive for coagulase-positive *S. aureus* and most of the cheese samples presented 4.00–5.00 log CFU/g. Additionally, total coliforms were reported in 53.3% of cheeses, 33.3% of samples were positive for *E. coli* and 60% of samples had *E. coli* > 2.00 log CFU/g, which can be considered as a potential health risk and an indicator for potential fecal contamination. These results for contaminants, high number of coagulase-positive bacteria and coliforms, indicated hygienic problems during production and/or storage.

The safety of the Canastra cheese is a serious issue, given the fact that it is produced with raw milk and very frequently by small-scale producers, sometimes even under home kitchen production conditions. Borelli et al. [31] assessed the population dynamics of *Staphylococcus* spp. during the ripening of the Canastra cheese in three different farms. The authors focused on the presence of the coagulase (*coa*), thermonuclease (*nuc*), and enterotoxin (*sea*, *seb*, *sec*, and *sed*) genes investigated in *Staphylococcus* strains isolated from the Canastra cheese during the 60 days of ripening. *Staphylococcus* spp. were found in samples ripened from 0 to 45 days, ranging from 10^3^ to 10^8^ CFU/g. All isolated *Staphylococcus* spp. were considered coagulase-positive based on the physiological tests that generated evidence for carrying the *coa* gene. The coagulase and thermonuclease genes occurred simultaneously in 41.3% of the tested *Staphylococcus* spp. The enterotoxins were not detected in any of the cheese samples and none of the investigated *Staphylococcus* strains expressed the staphylococcal enterotoxins.

*S. aureus* strains were isolated as well by Das Dores et al. [5], from the Canastra cheese produced in dry and rainy seasons, in different farms. Their ability to produce enterotoxins was explored through biochemical and bio-molecular approaches, targeting the classical enterotoxin genes. The staphylococcal enterotoxin A (*sea*) was detected in 75% of cheese samples, but no toxin was detected through the ELFA-VIDAS (BioMerieux, Marcy-l’Etoile, France) method. Moreover, 12.5% of isolated strains produced *sea* and staphylococcal enterotoxin C (*sec*). When using the PCR assay to target these genes, only one isolate was found to harbor an enterotoxin gene, contrary to expectations based on biochemical results. However, most probably, the studied *S. aureus* can carry different genes related to the protein expression, known as non-classical enterotoxins. High *S. aureus* counts in the cheese samples are a cause of concern since there is a risk of the presence of these non-classical enterotoxins and potential of health hazards.

The detection of *S. aureus* at levels above those permitted by law have frequently been reported for many types of Minas artisanal cheese, such as Canastra [5] and Serro [46]. The control of *Staphylococcus* spp. levels in cheese is considered as a marker for assessing the safety of dairy products. Outbreaks of food poisoning related to toxins (staphylococcal enterotoxins) produced by different *Staphylococcus* spp. were previously reported and were associated with the consumption of traditional artisanal cheeses in Brazil [72]. It is well known that some *Staphylococcus* strains can be responsible for food poisoning related to the production of enterotoxins.

Staphylococci contaminations are probably the most frequent problem regarding artisanal cheeses [32,47,73,74,75,76,77]. It is usually associated with the contaminations from the animals’ skin or employees directly involved in the milking process, or even the manipulation during cheese-making. Generally, authors associate microbial contamination in dairy products with the use of non-pasteurized milk; however, streptococci contaminations are responsible for the production of toxins, most of them heat-stable, so they are not affected by the pasteurization processes [5].

Aragão et al. [32] reported 54 *S. aureus* isolates found in artisanal Coalho cheese from 11 municipalities in the state of Pernambuco, which were characterized as positive for *bla*Z gene and methicillin-resistant, raising a concern for the health of the consumers of this type of cheese. Counts for coagulase-positive *Staphylococcus* were above the legal limits in many samples of Canastra artisanal cheese from the Serra da Canastra area [47]. Among the artisanal cheeses from the Northwestern region of Paraná analyzed by Castilho et al. [76], 85% presented counts above 10^3^ CFU/g for coagulase-positive staphylococci, most probably related to milk obtained from cows suffering from mastitis and due to the fact that most of the artisanal cheeses are made of raw milk; when it happens with pasteurized milk cheese, it is possibly as a consequence of an inadequate thermal process or post-processing recontamination [73]. Castro et al. [75] reported 76 staphylococcal isolates from Minas artisanal cheese from the area of Campo das Vertentes. A low expression of the staphylococcal toxin genes was detected according to the performed quantitative real-time PCR (qRT-PCR), and only 2 staphylococcal isolates produced toxic shock syndrome toxins. More than half of the evaluated staphylococcal isolates (56.58%) were identified with hemolytic activity, 67.11% of isolates were penicillin G, and 27.63% were tetracycline resistant [75]. Grecelle et al. [74] isolated 72 *Staphylococcus* sp. isolates from a Colonial cheese in the state of Rio Grande do Sul during different stages of the production process; among the *Staphylococcus* isolates, 43% were identified as coagulase-positive with predominance of *S. warneri* and *S. sciuri*. Moreover, *S. aureus* was identified as the predominant species in the raw milk and production tanks. Grecelle et al. [74] identified eleven different species of *Staphylococcus* present in the colonial cheese, with the predominant presence of *S. aureus*, recovered from different stages of the milk handling and cheese production process; this indicates serious hygienic issues to be addressed in that facility. Melo et al. [77] reported 58 coagulase-positive *Staphylococcus* and 46 coagulase-negative *Staphylococcus*, of which 33 were retrieved from raw milk and 71 from artisanal cheese produced in the Serrana area in the state of Santa Catarina. Among the 58 coagulase-positive *Staphylococcus*, the predominant ones were *S. aureus* (64%), *S. scheiferi* subsp. *coagulans* (22%), *S. hyicus* (12%), and *S. intermedius* (2%); the majority was positive for enterotoxins genes.

The presence of different pathogens in cheese is important to be evaluated during the entire process, starting from the raw material, to manufacturing and, especially, during the ripening period. Dalmina et al. [78] evaluated the artisanal Serrano cheese from Santa Catarina and throughout the 35 days of ripening. Samples from 14 farms still had counts above the limit defined in the legislation for *E. coli*, *Staphylococcus* spp., *L. monocytogenes*, and *Salmonella* spp. It is alarming that in one of the cheese facilities, the presence of *L. monocytogenes* was detected in all the evaluated periods, showing that ripening was not efficient to eliminate the pathogen [78]. Moreover, the presence of *L. monocytogenes* in cheeses from that same region had been previously reported, as shown by Melo et al. [79], when *L. monocytogenes* was found in three samples of artisanal Serrano cheese, isolated at 30 and 45 days of ripening. In a similar study, Pontarolo [80] identified *L. monocytogenes* in artisanal Serrano cheese at 14 days, and in two samples at 28 days of ripening. Campos et al. [47] reported one positive sample for *L. monocytogenes* in Canastra artisanal cheese from the Serra da Canastra area. It is well accepted that during the cheese ripening period, when there is a reduction of water activity and other changes take place, there is expected to be a reduction of food-borne pathogens. This is why the Brazilian legislation recommends that artisanal cheese made according to traditional procedures needs to be maturated and commercialized only after a specific period, a process directly related to food safety. However, *L. monocytogenes* is tolerant to high salt and acid concentrations and is able to survive at refrigeration temperatures, which represents a series of obstacles for its easy control in fermented food products [81].

Apart from the previously reported contaminations, some reports are pointing to other relevant microbiological problems during the cheese production and presence of contaminants, which can have health consequences for consumers. Albuquerque et al. [43] reported an occurrence of *Mycobacterium avium* subsp. *paratuberculosis* in the Coalho cheese produced in the state of Pernambuco, Northeastern Brazil. Normally, this pathogen is involved in the paratuberculosis of ruminants or other domestic animals; however, it may also be associated with some humans’ clinical conditions, such as Crohn’s disease, type 1 diabetes, Hashimoto’s thyroiditis, sarcoidosis [82,83,84,85,86]. Forty samples of Coalho cheese produced with non-pasteurized cow’s milk were analyzed; 27.5% (11 cheeses) were found to be positive for *M. avium* subsp. *paratuberculosis*, based on qPCR.

Medeiros et al. [18] reported an analysis of 50 different samples of Coalho cheese from the Northeastern regions of Brazil in which they were screened for the presence of different representatives of mycobacteria based on conventional and RT nested PCR. Even though all studied cheese samples were negative for *M. tuberculosis*, two cheeses (both artisanally produced with raw milk) showed positive evidence for the presence of *Mycobacterium lehmanii* and *Mycobacterium rutilum*, most probably as a result of environmental contamination [18].

Moreover, Albuquerque et al. [43] and Medeiros et al. [18] detected the DNA of mycobacteria (*M. avium* subsp. *paratuberculosis*, *M. lehmanii*, and *M. rutilum*), and not the bacterial cells of the pathogen itself; also, they did not provide evidence that showed if the DNA was from dead or living cells. However, even if only dead DNA cells were detected and not living pathogens were present in the food product, it is enough evidence of a serious problem in the production of this specific cheese, resulting in contamination of the production line or sick animals in the supply chain for that cheese production facility. It is a serious problem that requires greater attention on hygiene during the cheese production, not only related to Good Manufacturing Practices (GMP), but also to Good Agricultural Practices (GAP).

Additionally, reports on Q fever/coxiellosis are alarming, since they can be related to cheese as well [87]. From a global perspective, increase in Q fever research was recorded after the 2007 epidemic in the Netherlands, whereas in Brazil, the epidemiology of coxiellosis is still poorly understood and not very well mapped. Rozental et al. [87] researched, using the PCR approach, the prevalence of *C. burnetii* in Minas artisanal cheeses from the Serro area by extracting the DNA from 53 cheese samples. An analysis was performed by means of nested species-specific PCR for *C. burnetii* and the obtained amplicons were sequenced. Only 9.43% of the analyzed cheeses had DNA-positive *C. burnetii* [87].

Cheese contaminations can be related to poor hygiene procedures during milk production (milking, animals’ and workers’ health conditions, ineffective equipment cleaning, both inadequate handling and storage), as well as lack of GMP during cheese production. In this context, the artisanal Minas cheese, such as the Canastra cheese, can be considered as a well studied cheese from the microbiological point of view, followed by the Serro cheese. Additionally, if we considered the presence of pathogenic bacteria, hygienic indicators, starter culture, beneficial bacteria, and the knowledge about the presence of yeast, it has even more to be explored. Furthermore, the microbiological quality and safety of other artisanal Brazilian cheeses are almost rare.

In general, artisanal cheese producers need to be conscious and trained on basic knowledge of food hygiene, food contamination, food safety, as well as cheese technology, in order to reduce the levels of food-borne illnesses associated with cheese and to improve the product quality. Furthermore, complementary studies are also necessary to deeply understand the relationship between technological processes and microbiological properties, which result in higher culinary quality and safety of the artisanal Brazilian cheese.

## 6. Conclusions

Artisanal Brazilian cheese, and particularly artisanal Minas cheese, is a highly appreciated dairy product from specific regions of Brazil. For more than 200 years, some artisanal Brazilian cheese products have been made under semi-industrial or artisanal manufacturing conditions with raw milk, which results in these typical cheeses. The cheeses go through different technological processes and have unique characteristics. Even if some of the Brazilian cheeses are produced using similar technological processes, such as Minas artisanal cheese, since they are produced with raw milk and whey from previous batches (for some cheeses, *pingo* or *rala*) as a natural starter, the cheese sensory characteristics reflect their “terroir” and they are known by the region or city where they are produced. Additionally, the different Brazilian regions present specific conditions for ripening, such as humidity, temperature, and indigenous microbiota which affect cheese characteristics.

The controversial question remains about the culinary quality and safety of the products. The crucial point is that producers need to introduce SOP and GMP in their production, and preferentially obtain certain knowledge on food microbiology in order to guarantee the market expansion of a good-quality and safe food product.

## Figures and Tables

**Table 1:** General characteristics of other Brazilian artisanal cheeses.

Cheese Name	Region and State	Type of Milk	Size and Shape	ManufacturingProcess and Classification	General Characteristics and Ways of Consumption	References
Artisanal Minas	Cerrado, MG	Raw cow’s milk	Cylindrical, 15–17 cm diameter, 4–6 cm height and 1–1.2 kg	Semi-hard cheese produced using fermented whey as a natural starter and rennet for coagulation. The curd is cut, stirred, molded, salted, and ripened for at least 22 days. It is classified as uncooked, pressed and short-ripened cheese.	It presents yellowish fine rind, without cracks, the curd is yellowish-white, firm texture, semi-hard and buttery consistency; the taste is slightly acid, not pungent, and pleasant.	[1,53]
Artisanal Minas	Alagoa, MG	Raw cow‘s milk	Cylindrical, from 1 to 5 kg	Inspired by Parmesan cheese. It is classified as cooked, pressed, natural rind and short-ripened cheese.	Heated and pressed curd and ripened for 7 days to 2 months for 1 kg, and 2 years for 5 kg.	[1]
Alpino	Corumbá de Goiás, GO	Pasteurized cow’s milk	Cylindrical; the weight varies from 0.4 to 5 kg.	Swiss-type cheese, classified as cooked and pressed.	It presents a typical dry fruit and herb aroma. The taste is slightly spicy and intense, has low salt, and the texture is firm and creamy.	[27]
Boursin de cabra	Rochedinho, MS	Pasteurized goat’s milk	Small balls with herbs and pepper immersed in olive oil in jars of 200 g.	French-origin cheese produced by rennet coagulation and LAB cultures, draining the curd, adding dairy cream and garlic, chimichurri (a traditional mixture of seasonings and peppers) and Provence herbs, and molding. It is classified as fresh cheese.	It presents a soft, creamy and spreadable texture; the color is white and without rind, the taste is mild, buttery and fresh. It is consumed as an appetizer or as an ingredient for salads.	[27]
Braz Mato-gros- sense	Cuiabá, MT	Raw cow’s milk		Inspired by Italy, cooked and pressed cheese. Three versions of ripening—fresh: 2 months, mezzano: 6 months, and gran riserva: 12 months.	It presents mild lactic, buttery and fresh herb taste. This cheese is consumed as an appetizer or used as an ingredient in Italian dishes.	[27]
Zola Mato-gros- sense	Cuiabá, MT	Raw milk		The cheese is classified as soft blue cheese (*Penicillium roqueforti*) with washed rind.	It is a creamy cheese, slightly spicy. The aroma recalls mushrooms and rural areas. It is commonly consumed as an appetizer.	[1,27]
Azul do Bosque	Joanópolis, SP	Pasteurized goat’s milk	Cylindrical 200 g	Semi-hard blue cheese ripened during 40 to 50 days by internal molds. The *Penicillium roqueforti* grows inside and outside the cheese.	The cheese was inspired by the English Stilton cheese. It has a distinguished spicy taste, with a herb and mushroom aroma. It is commonly consumed as an appetizer.	[1,27]
Coração em Brasa	Joanópolis, SP	Pasteurized goat’s milk	Heart shape with 120 g.	Soft cheese ripened by white mold over coverage of vegetable charcoal for 20 to 30 days. The curd is lightly pressed and filled with a thin layer of smoked chipotle, habanera and cayenne pepper.	The spices confer light acid, intense and spicy taste, and fermented dairy aroma. This cheese can be a component of a cheese plate appetizer or consumed with toast or bread as a starter menu.	[27]
Embriago d’Alagoa	Alagoa, MG	Raw cow’s milk	Cylindrical	Inspired by semi-hard Belgian cheese and washed with beer. Ripened for 40 days and washed with Jaboticaba (*Plinia cauliflora*) liqueur. Presents a rind of *Brevibacterium linens* and white mold.	The action of *Brevibacterium linens* and white mold during ripening changes the curd into a creamy texture and aromatic flavor. This cheese is consumed as an appetizer.	[27]
D’Alagoa Faixa Dourada	Alagoa, MG	Raw cow’s milk	Cylindrical and 1.0 kg	Cheese is obtained by pressing and heating the curd and ripening for 60 days.	The taste is aromatic, pungent, persistent, and slightly salty. The consistencyis soft.	[1]
Cabacinha	Pedra Azul, Cachoeira do Pajeú, Comercinho, Medina and Itaobim, MG	Raw cow’s milk	Calabash shape	The cheese is similar to the Italian Caciocavallo cheese. It is classified as a pasta filata cheese.	The cheese is stored unpackaged and at room temperature. Can be smoked or filled with butter. It is consumed as an appetizer.	[54]

MG: Minas Gerais; MS: Mato Grosso do Sul; MT: Mato Grosso; GO: Goiás; SP: São Paulo states.

## Data Availability

Not applicable.
